# Religion, Spirituality, and Well-Being: An Underexplored Aspect of Growing Older in the United Kingdom?

**DOI:** 10.1093/geroni/igaa057.2358

**Published:** 2020-12-16

**Authors:** Christina Victor, Ruth Lamont, Isla Rippon, Linda Clare

**Affiliations:** 1 Brunel University London, Uxbridge, England, United Kingdom; 2 University of Exeter, Exeter, England, United Kingdom

## Abstract

There is a rich literature from The United States looking at the importance of religion and spirituality in the lives of older adults where it is positively linked with wellbeing. Despite the increased interest in wellbeing in the UK comparatively little interest has been show in the role of religion and spirituality in promoting wellbeing including quality of life, life satisfaction and loneliness. In this paper we explore these issues using three data sets: the European Social Survey (ESS), the English Longitudinal Study of Ageing (ELSA) and the IDEAL cohort of people with dementia and their carers to examine (a) the variation in religious practice by older adults, those aged 50+, across Europe; (b) the epidemiology of religious practice among older adults within England and (c) using both ELSA and IDEAL consider the relationship between religion and wellbeing in later life.

